# Investigating the risk of reintubation by cough force assessment using cough peak expiratory flow: a single-center observational pilot study

**DOI:** 10.1186/s12890-024-02914-0

**Published:** 2024-05-07

**Authors:** Kenya Murata, Keiichiro Shimoyama, Takeshi Tsuruya

**Affiliations:** 1 Department of Nursing, Tokyo Medical University Hospital, Tokyo, Japan; 2 Department of Emergency and Critical Care Medicine, Tokyo Medical University Hospital, Tokyo, Japan

**Keywords:** CPEF, Ventilator, SBT, Extubation, Reintubation, Cough, ICU

## Abstract

**Background:**

No objective indicator exists for evaluating cough strength during extubation of tracheally intubated patients. This study aimed to determine whether cough peak expiratory flow (CPEF) can predict the risk of reintubation due to decreased cough strength.

**Methods:**

This was a retrospective cohort study of patients who were admitted to our Emergency Intensive Care Unit between September 1, 2020 and August 31, 2021 and were under artificial ventilation management for ≥ 24 h. The patients were divided into two groups: successful extubation and reintubation groups, and the relationship between CPEF immediately before extubation and reintubation was investigated.

**Results:**

Seventy-six patients were analyzed. In the univariate analysis, CPEF was significantly different between the successful extubation (90.7 ± 25.9 L/min) and reintubation (57.2 ± 6.4 L/min) groups (*p* < 0.001). In the multivariate analysis with age and duration of artificial ventilation as covariates, CPEF was significantly lower in the reintubation group (*p* < 0.01). The cutoff value of CPEF for reintubation according to the receiver operating characteristic curve was 60 L/min (area under the curve, 0.897; sensitivity, 78.5%; specificity, 90.9%; *p* < 0.01).

**Conclusion:**

CPEF in tracheally intubated patients may be a useful indicator for predicting the risk of reintubation associated with decreased cough strength. The cutoff CPEF value for reintubation due to decreased cough strength was 60 L/min.

## Introduction

Many patients in the emergency and intensive care fields are treated with invasive mechanical ventilation. In the United States, 29% of patients admitted to intensive care units (ICUs) are on ventilators [1], and in Japan, 24% of patients admitted to ICUs are on ventilators [2]. Prolonged ventilator management leads to ventilator-associated pneumonia (VAP), which is reported to occur in 9–27% of all intubated patients [3]. Furthermore, VAP can result in a longer hospital stay of 4.3 days and a mortality rate of 32% [4–6]. Recently, spontaneous breathing trials (SBTs) have been recommended as a measure of breathing to determine whether extubation is successful or not [7]. Even in patients successfully extubated with SBT, approximately 10–25% of patients are reintubated, and when reintubation does occur, the mortality rate is reported to be high [8–10]. Reintubation may be due to respiratory insufficiency, which is a cause of death or disability. Although many causes of reintubation have been reported, including respiratory failure, circulatory failure, and decreased level of consciousness, the most common cause of reintubation after extubation is upper airway problems, with difficulty expectorating airway secretions reported to be the most common [11, 12]. Therefore, properly assessing the coughing ability of ventilator patients and considering the risk of reintubation are extremely important.

It has been reported that cough peak expiratory flow (CPEF) is useful as an index for evaluating cough strength and reflecting the expectorant capacity of nonintubated patients [13–15]. This can measure the intensity of voluntary coughing using a peak flow meter and is a simple and objective way to evaluate coughing ability. Although CPEF can be measured in intubated patients depending on the ventilator, studies evaluating its usefulness are extremely limited. In previous studies [16–18] and a recent systematic review [19], the predictive power of extubation failure using CPEF has been examined, and its correlation with reintubation, which indicates its potential utility, has been reported. However, these studies included factors other than cough strength, such as laryngeal edema and circulatory failure, in their definition of reintubation, thus leading to doubt on whether CPEF accurately reflects cough strength. Consequently, it remains unclear whether CPEF can serve as a reliable indicator of cough strength in intubated patients at this point.

Therefore, this study aimed to evaluate the association between CPEF and reintubation due to decreased coughing ability in intubated patients.

## Methods

### Study design and settings

This study was a single-center retrospective study conducted at Tokyo Medical University Hospital from September 1, 2020 to August 31, 2021. The study was approved by the Ethics Committee of Tokyo Medical University (T2021-0221). The ethical standards of the Declaration of Helsinki and the Strengthening the Reporting of Observational Studies in Epidemiology checklist were followed in this study. Disclosure of information and opt-out forms of informed consent were used.

### Study participants

Patients admitted to the Emergency Intensive Care Unit who were on ventilator management for > 24 h and who successfully underwent SBT were included in this study. Of these patients, those who were transferred to a different floor or hospital with oral intubation without extubation, pediatric patients under the age of 18 years, and patients with missing records were excluded. Patients with tracheostomy were also excluded. The definition of reintubation was based on previous studies [20] and was defined as cases of reintubation within 72 h after extubation, excluding cases of reintubation due to causes unrelated to coughing power, such as vocal cord paralysis, laryngeal edema, and circulatory failure. Causes of reintubation were extracted from the medical records of physicians in the electronic medical records. The ventilator of the patients was limited to a Puritan Bennett 980 (Medtronic, Minneapolis, MN, USA) to visually measure CPEF from the graphic waveform, referring to a previous study [20].

### Measurements/exposures/candidate predictors

The background characteristics of the patients were extracted from their medical records: sex, age, weight, Acute Physiology and Chronic Health Evaluation (APACHE) II score, duration on the ventilator, CPEF before extubation, methods of respiratory management after extubation, primary illness, and whether the patient was reintubated.

#### Measurement of CPEF

**Fig. 1:**
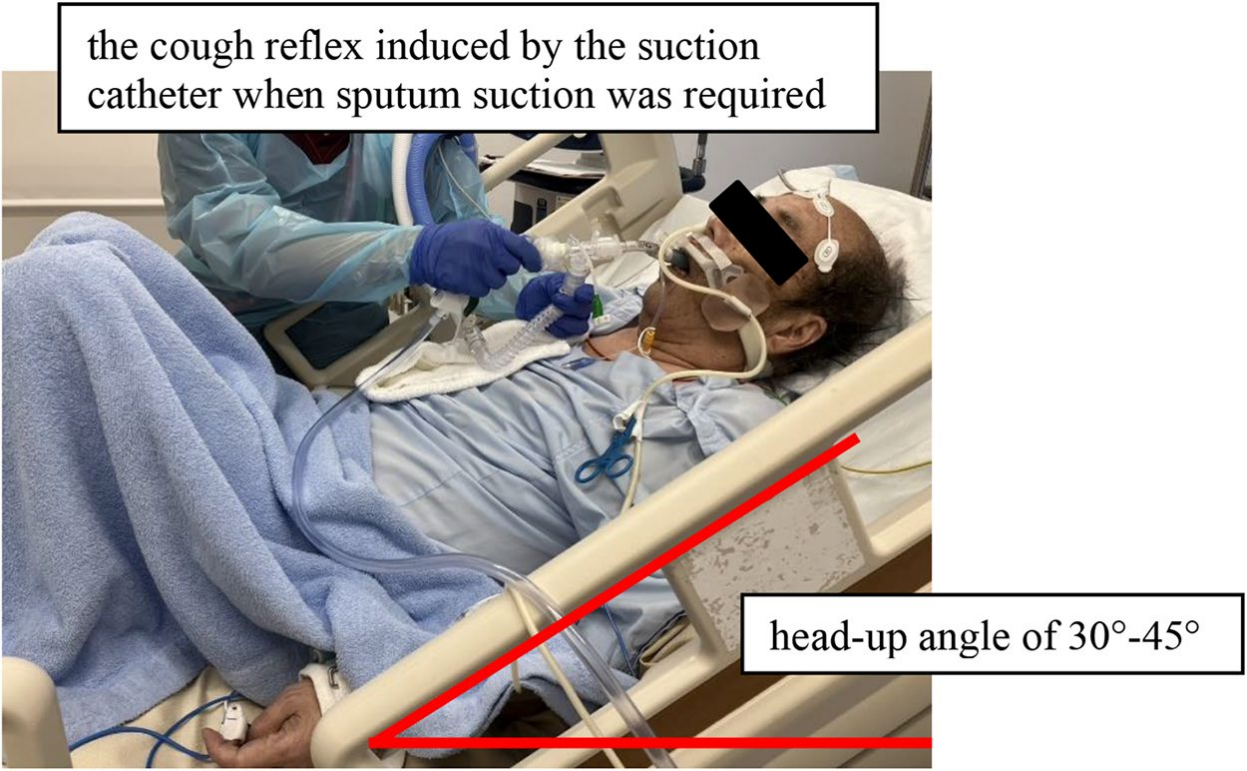
CPEF measurement method. From the cough reflex elicited by the suction catheter when suction was required, the highest expiratory flow velocity in the flow waveform at that time was read and visually recorded by two nurses from the graphic waveform and used as the CPEF

**Fig. 2:**
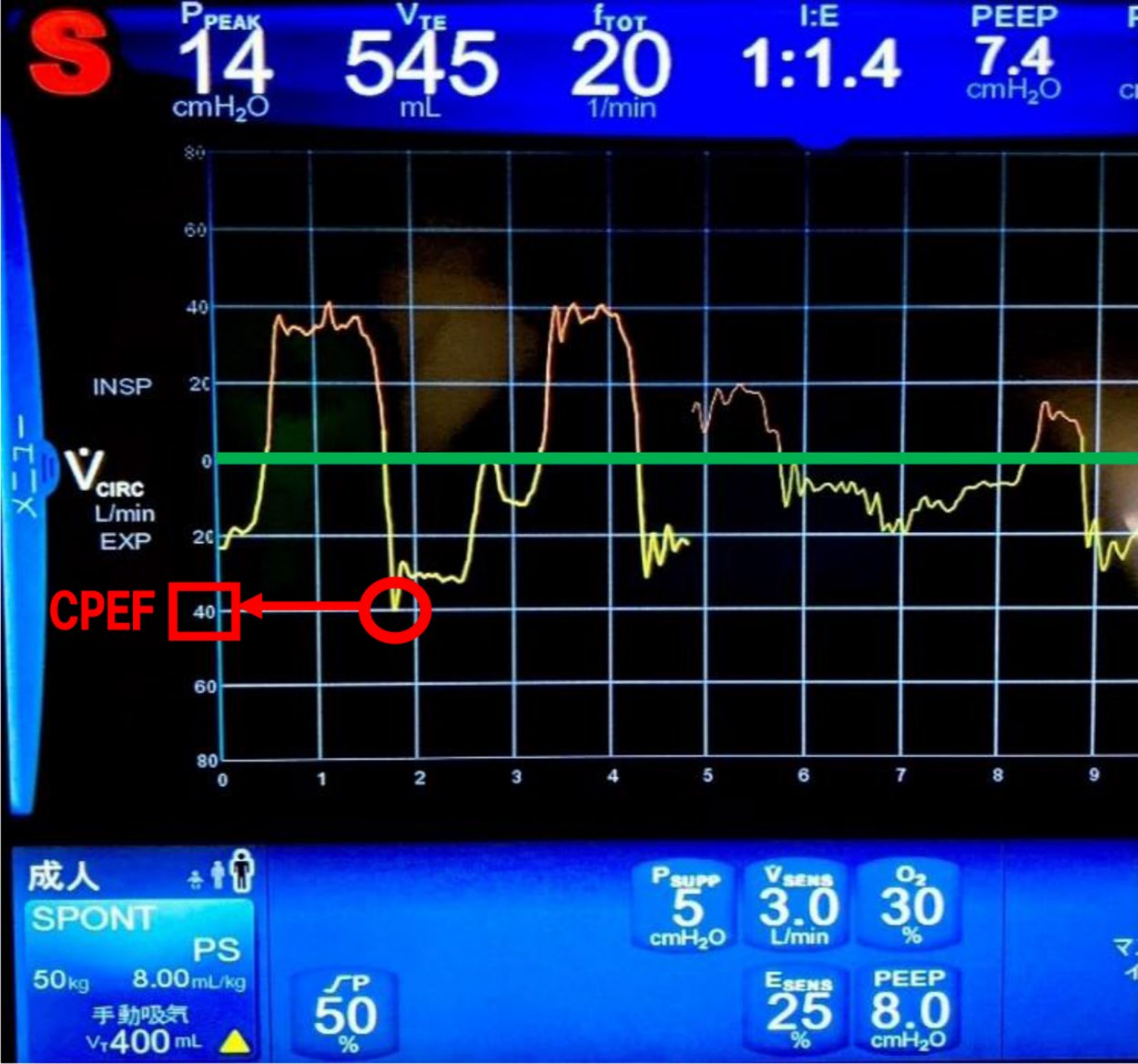
How to read graphic waveforms. Graphic waveform of flow rate-time. The vertical axis represents the flow rate (L/min), whereas the horizontal axis depicts time (seconds). The green line shows the baseline, and below the baseline is the exhalation. The maximum flow rate of exhaled air was visually read from the graph and used as data

Measurement Method of CPEF (see Fig. 1): The CPEF of the cough reflex induced by the suction catheter when sputum suction was required was read from the flow waveform of the ventilator’s graphic monitor [21]. Figure 2 shows the method for interpreting the graphic waveform. Values were visually confirmed by two nurses and read from the vertical axis of the expiratory flow waveform. The highest CPEF value induced during sputum suctioning was used as data. Referring to a previous study [22], the minimum unit of measurement was 10 L/min at 60 L/min or higher and 5 L/min at 40 L/min due to the flow waveform scale at this time. CPEF was measured using a uniform method in all cases, with no specific method changes depending on the disease. CPEF has been reported to be a factor influenced by posture at the time of measurement, the measurement method was consistently performed at a uniform head-up angle of 30°–45°. The ventilator mainly used at our hospital is Puritan Bennett 980 (Medtronic, Minneapolis, MN, USA); therefore, this study was limited to the application of PB980. Respiratory management at the time of extubation was primarily conducted using a Venturi mask and low-flow oxygen device.

Ventilator settings: In all patients, SBTs were performed according to the American Association for Respiratory Care guidelines [23] with a reduced dose of analgesic and sedative medications and a change in setting to pressure support ventilation after assessing the presence of spontaneous respiration. The patients were evaluated with pressure support ventilation ≤ 5–7 cmH2O, positive end-expiratory pressure ≤ 5cmH2O, and fraction of inspired oxygen ≤ 0.4, and extubation was considered based on comprehensive judgment by the physician and nurses.

In a previous study [24], comparing CPEF using three cough stimulation methods, no statistically significant difference in CPEF was observed among the methods, indicating high diagnostic accuracy for the success or failure of extubation. Because it has been highlighted that measuring CPEF by voluntary coughing is inaccurate depending on the level of consciousness and degree of patient cooperation, CPEF was measured using the cough reflex in this study.

### Outcomes

We validated the validity of a secondary evaluation item, namely, the cutoff value of CPEF leading to reintubation, using the presence or absence of reintubation as the main evaluation item.

### Statistical analysis

We performed a comparative analysis of two groups: a successful extubation group and a reintubation group. For categorical variables, we used the chi-squared test or Fisher’s exact test, whereas, for continuous variables, we used the t-test and Mann–Whitney U test. There are multiple confounding factors for the risk of reintubation, such as age, P/F ratio, APACHE II score, weight, and duration of mechanical ventilation, in addition to CPEF. Therefore, we investigated the association between CPEF and the presence of reintubation using univariate and multivariate logistic regression analyses. In the multivariate analysis, we adjusted for age and duration of mechanical ventilation as covariates, as in previous studies [25, 26].

We used R (version 1.55) for all statistical analyses. All statistical tests were two-sided, and a significance level of 5% was used to evaluate the *p*-values for each evaluation item.

To further investigate the relationship between the successful extubation group and the reintubation group, we used receiver operating characteristic (ROC) curve analysis and calculated a cutoff value for predicting reintubation due to weak cough reflex. The cutoff value was determined as the threshold closest to the upper left corner of the ROC curve, and we evaluated its predictive performance using the positive and negative predictive values at the moderate level.

## Results

**Fig. 3:**
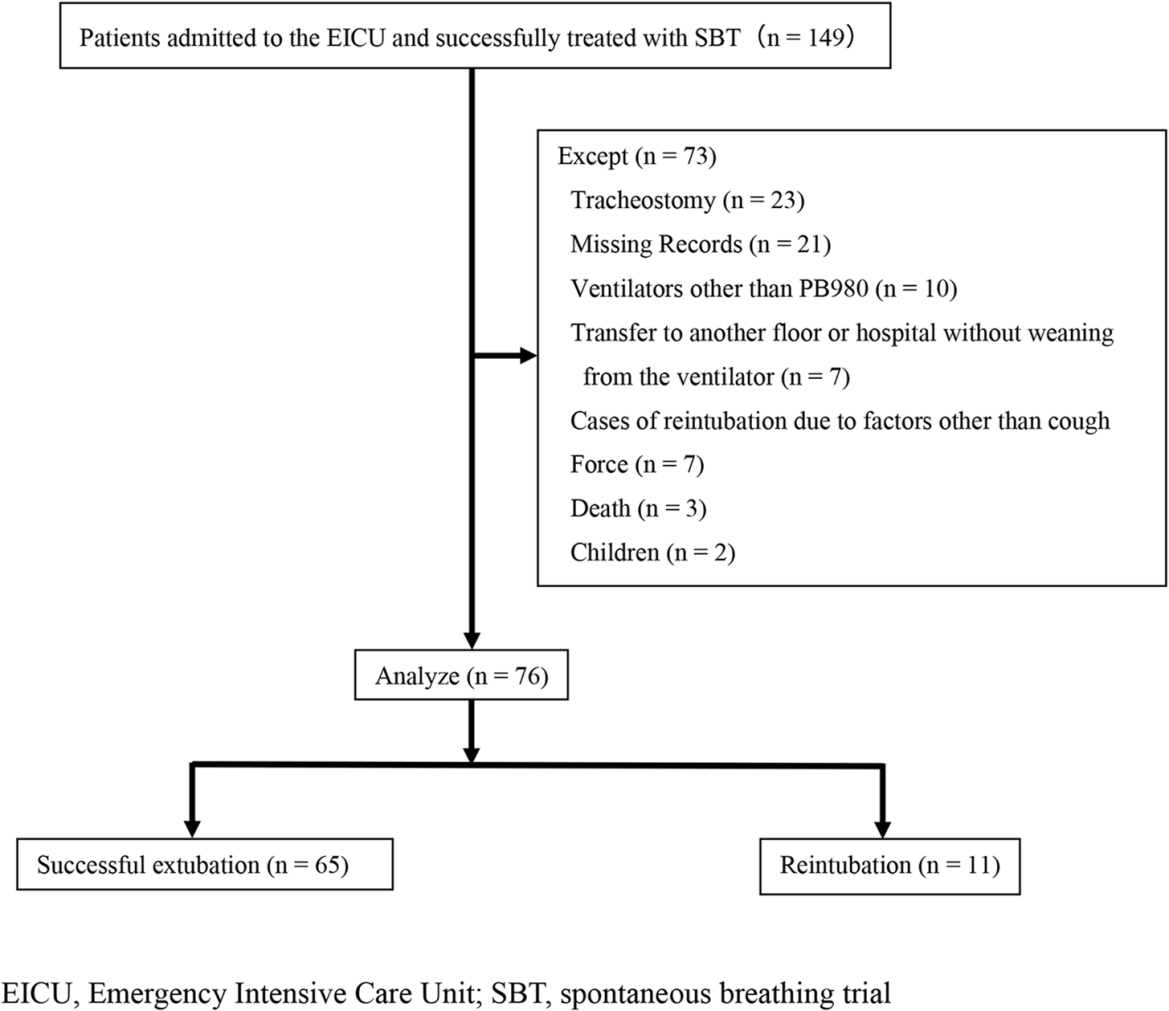
Study flowchart

Figure 3 shows the study flowchart. Of the 76 cases eligible for the study, 65 were in the extubation success group and 11 were in the reintubation group, after excluding ineligible cases. Table 1 shows the background characteristics of the patients. No significant differences in the background characteristics were observed between the two groups. We found no significant difference in the methods of respiratory management after extubation (P-value = 0.656). However, a significant difference in CPEF was observed between the extubation success group (90.7 ± 25.9 L/min) and the reintubation group (57.2 ± 6.4 L/min) (*p* < 0.001).

**Table 1:** Patient characteristics

	Total	Extubation	Extubation failure	*P*-value
	(*n* = 76)	(*n* = 65)	(*n* = 11)	
Sex(male/female)	50/26	43/22	7/4	0.879
Age, years	56	56(18–87)	60(42–80)	0.352
Weight (kg)	64	65(29–90)	54(41–82)	0.298
APACHE II	16.0 ± 7.4	15.8 ± 7.4	17.4 ± 7.1	0.506
The duration of MV(days)	7.2 ± 4.9	6.8 ± 4.5	9.8 ± 6.5	0.061
CPEF(L/min)	85.9 ± 26.8	90.7 ± 25.9	57.2 ± 6.4	< 0.001
HFNC after extubation	11	9	2	0.656
**Diseases for hospitalization(%)**			0.796	
Cardiovascular disease	4(5%)	4(6%)	0(0%)	
Respiratory disease	30(39%)	27(41%)	3(27%)	
Digestive disease	11(14%)	9(13%)	2(18%)	
Severe trauma	12(15%)	10(15%)	2(18%)	
Nervous system disease	13(17%)	10(15%)	3(27%)	
Mental disease	1(1%)	1(1%)	0(0%)	
Metabolic disease	5(6%)	4(6%)	1(9%)	

Sex and diseases for hospitalization were analyzed using Fisher’s exact test

Age and weight are expressed as medians using the Mann–Whitney’s U test.

APACHE II score, duration of MV, and CPEF are expressed as means ± standard deviations using the t-test

APACHE II, Acute Physiological Assessment and Chronic Health Evaluation II; MV, mechanical ventilation; CPEF, cough peak expiratory flow

HFNC, High Flow Nasal Cannula

Tables 2 and 3 show the results of the univariate and multivariate logistic regression analyses, respectively. The odds ratios for CPEF were 0.87 (95% confidence interval [CI], 0.794–0.958) and 0.85 (95% CI, 0.770–0.949), respectively, and were statistically significantly associated with reintubation (*p* < 0.05).

**Table 2:** Factors affecting extubation failure results of the univariate logistic regression analysis

Variable	Odds ratio	95% confidence interval	*P*-value
CPEF(L/min)	0.87	0.794–0.958	0.004

The model was calculated using univariate logistic regression. Success or failure of extubation was determined as the dependent variable, and CPEF, duration of MV, and age were determined as independent variables.

CPEF, cough peak expiratory flow; MV, mechanical ventilation.

**Table 3:** Factors affecting extubation failure results of the multivariate logistic regression analysis

Variable		Odds ratio	95% confidence interval	*P*-value
CPEF(L/min)		0.85	0.770–0.949	0.003
The duration of MV(days)		1.30	1.010–1.670	0.041
Age		1.04	0.970–1.200	0.262

The model was calculated using multivariate logistic regression. Success or failure of extubation was determined as the dependent variable, and CPEF, duration of MV, and age were determined as independent variables

CPEF, cough peak expiratory flow; MV, mechanical ventilation

**Fig. 4:**
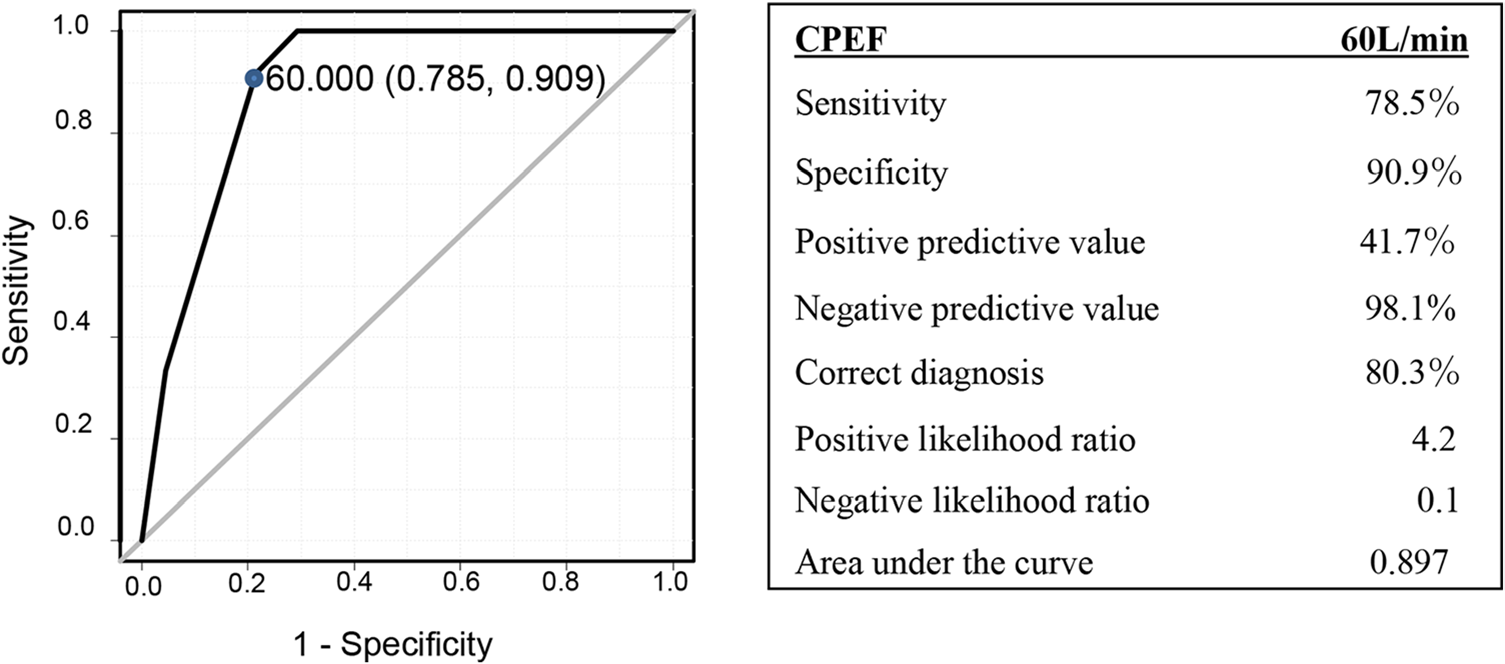
The ROC and curve of CPEF. The ROC curve was used to calculate the AUC (SE) and the cutoff value to predict reintubation due to reduced coughing capacity. CPEF, cough peak expiratory flow; AUC, area under the ROC curve; ROC, receiver operating characteristic

Figure 4 shows the ROC curve of CPEF for reintubation. The cutoff value of CPEF as a predictor of reintubation was 60 L/min, with a sensitivity of 78.5% and specificity of 90.9%. The area under the ROC curve was 0.897 (95% CI, 0.831–0.964). The negative and positive predictive values of CPEF were 98.1% and 80.3%, respectively, and the negative likelihood ratio was 0.1

## Discussion

In this study, CPEF was statistically significantly associated with reintubation due to decreased coughing ability in patients successfully weaned from mechanical ventilation with SBT. Moreover, a CPEF cutoff value of 60 L/min was determined for reintubation, suggesting that CPEF is useful in predicting reintubation due to decreased coughing ability. Salam et al. [27] have investigated the relationship between airway physiology and extubation success in 88 mechanically ventilated patients who successfully passed an SBT by evaluating the cough peak flow, properties of airway secretions, and responses to four commands (i.e., opening eyes, following gaze, shaking hands, and sticking out tongue). The results have shown that if the three aforementioned factors were positive, extubation would fail 100% of the time, implying a correlation between CPEF and extubation success. Studies [20] have also concluded that CPEF was an indicator of coughing ability and a factor related to reintubation, with CPEF values of 80 ± 26 L/min in the successful extubation group and 50 ± 22 L/min in the reintubation group and a CPEF cutoff of 56 L/min for reintubation. However, both studies [16–19] have included reintubation cases that were unrelated to decreased coughing ability, such as laryngeal edema and circulatory failure; therefore, CPEF did not directly demonstrate the association with coughing ability. In this study, reintubation cases were limited to those due to decreased coughing ability; thus, the possibility of a relationship between CPEF and reintubation due to decreased coughing ability was suggested.

Based on the results of this study, when considering extubation, it is possible that a CPEF ≤ 60 L/min reflects insufficient cough strength, which may increase the risk of reintubation due to difficulty in sputum expectoration. Therefore, care should be taken when increasing CPEF. It has been reported [28] that CPEF can be increased by step-up positioning, such as the supine position, 45° sitting, sitting at the edge of the bed, and ambulation, which are factors that can affect CPEF, in addition to posture, respiratory muscle strength, and chest expansion [21]. Therefore, advancing ambulation can be considered a care. Additionally, CPEF has been reported to be affected by pain and sedatives [29]. Thus, considering measures to address the factors causing low CPEF is necessary, such as pain control or delaying extubation time, if prolonged use of sedatives is suspected.

Similar to our study, Su et al. [16] have measured CPEF during cough reflex using an electronic flowmeter in patients admitted to ICUs who completed SBT and reported a cutoff value of 58.5 L/min as being associated with a higher risk of extubation failure. However, using a spirometer requires the addition of new equipment and training in measurement methods, which create barriers to generalization. The CPEF measurement method used in our study involved visually reading graphic waveforms from a mechanical ventilator, was noninvasive and simple, and can be easily performed by various healthcare professionals, including nurses, making it widely applicable in clinical settings.

In this study, CPEF was useful for predicting the risk of extubation failure due to decreased cough reflex and could aid in the objective assessment of cough strength in intubated patients. The numerical quantification of cough strength in patients may facilitate information sharing among healthcare professionals, including physicians and nurses, and contribute to reducing the rate of reintubation.

In this study, we excluded cases of reintubation due to factors other than cough strength in order to examine the relationship between CPEF and coughing ability. However, one of the limitations of our study is that as we included limited cases of reintubation, there is a potential of overestimation of the impact of cough strength; hence, caution is needed in generalizing the results. Furthermore, as CPEF measurements were visually interpreted, there is a possibility of bias in the numerical readings; thus, further investigation is warranted.

## Conclusion

It has been suggested that CPEF serves as an indicator of cough strength and a predictor of the risk of reintubation in patients with tracheal intubation. Furthermore, it is desirable to attempt extubation while considering the risk of reintubation associated with decreased cough strength, particularly when CPEF is below 60 L/min, and to provide necessary care accordingly.

## Data Availability

The datasets generated during and/or analyzed during the current study are available from the corresponding author on reasonable request.
